# Exploiting chemical ecology to manage hyperparasitoids in biological control of arthropod pests

**DOI:** 10.1002/ps.5679

**Published:** 2019-12-04

**Authors:** Antonino Cusumano, Jeffrey A Harvey, Mitchel E Bourne, Erik H Poelman, Jetske G de Boer

**Affiliations:** ^1^ Laboratory of Entomology Wageningen University Wageningen The Netherlands; ^2^ Department of Terrestrial Ecology Netherlands Institute of Ecology (NIOO‐KNAW) Wageningen The Netherlands; ^3^ Department of Ecological Sciences, Section Animal Ecology VU University Amsterdam Amsterdam The Netherlands

**Keywords:** push‐pull, infochemical‐based strategies, fourth trophic level organisms, hyperparasitoid foraging, multitrophic interactions, herbivore‐induced plant volatiles

## Abstract

Insect hyperparasitoids are fourth trophic level organisms that commonly occur in terrestrial food webs, yet they are relatively understudied. These top‐carnivores can disrupt biological pest control by suppressing the populations of their parasitoid hosts, leading to pest outbreaks, especially in confined environments such as greenhouses where augmentative biological control is used. There is no effective eco‐friendly strategy that can be used to control hyperparasitoids. Recent advances in the chemical ecology of hyperparasitoid foraging behavior have opened opportunities for manipulating these top‐carnivores in such a way that biological pest control becomes more efficient. We propose various infochemical‐based strategies to manage hyperparasitoids. We suggest that a push‐pull strategy could be a promising approach to ‘push’ hyperparasitoids away from their parasitoid hosts and ‘pull’ them into traps. Additionally, we discuss how infochemicals can be used to develop innovative tools improving biological pest control (i) to restrict accessibility of resources (e.g. sugars and alternative hosts) to primary parasitoid only or (ii) to monitor hyperparasitoid presence in the crop for early detection. We also identify important missing information in order to control hyperparasitoids and outline what research is needed to reach this goal. Testing the efficacy of synthetic infochemicals in confined environments is a crucial step towards the implementation of chemical ecology‐based approaches targeting hyperparasitoids. © 2019 The Authors. *Pest Management Science* published by John Wiley & Sons Ltd on behalf of Society of Chemical Industry.

## Graphical abstract

Insect hyperparasitoids can disrupt biological pest control by suppressing the population of their parasitoid hosts. In this review, we propose different infochemical‐based strategies that can be used to manage hyperparasitoids.

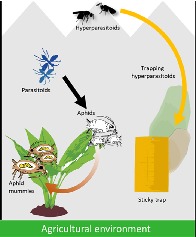

## 1 INTRODUCTION

### 1.1 Importance of parasitoids in biological control

Parasitoids are insects whose larvae develop in or on the bodies of other arthropods (mostly other insects), whereas the adults are free living. Most parasitoids are quite specialized and thus only attack a limited number of related species of hosts. Moreover, parasitoids generally attack a very limited number of host stages in nature, with different parasitoid species occupying well‐defined ‘guilds’.^1^ For instance, parasitoid guilds include specialized egg, egg‐larval, larval, larval‐pupal, pupal, and adult parasitoids. Because they are much more specialized than most predators, parasitoids have been extensively used in biological control programs for over a century, with often quite spectacular success both in open field and protected cropping systems.^2^ Indeed, today, parasitoids are of tremendous importance in biological control of arthropod pests worldwide.^3^ Well over 100 different species are commercially available for biological control of pest insects, making hymenopteran parasitoids the most diverse group of arthropod biocontrol agents. For example, *Trichogramma* species that parasitize the eggs of lepidopteran pests are released over millions of hectares to protect field crops such as corn, cotton and sugarcane. Likewise, parasitoids are very effective biocontrol agents of pest insects on ornamental or vegetable crops in greenhouses, including *Encarsia* spp. against whiteflies and *Aphidius* spp. against aphids. In their native ranges, naturally occurring parasitoids are also important in controlling herbivore populations, especially in cropping systems that are highly prone to pest infestations from native herbivores. Plant defenses are often much lower in crop plants compared with their progenitors because of domestication where certain traits, such as growth and taste, are selected at the expense of defenses.^4^ These factors make top‐down control of insect pests by natural enemies of paramount importance in cropping systems.

### 1.2 Hyperparasitoids are specialized natural enemies of parasitoids

Although parasitoids clearly mediate trophic cascades in natural and agro‐ecosystems by reducing herbivore abundance, food webs do not stop at three trophic levels and many contain four or even more.^5^ Indeed, parasitoids themselves have their own specialized natural enemies, i.e. so‐called hyperparasitoids, which can greatly hamper their effectiveness. Hyperparasitoids can be classified according to different life history traits.^6^ Based on a dichotomy in the way they exploit host tissues, two types of hyperparasitoids are distinguished. Primary hyperparasitoids are generally koinobionts (i.e. parasitoids that allow their host to continue development during parasitism)^7^ and parasitize immature primary parasitoid stages inside the body of their herbivore hosts.^6^ Secondary hyperparasitoids are predominantly idiobionts, i.e. parasitoids that arrest host development during parasitism,^7^ which attack non‐growing host stages such as pre‐pupae and pupae of their primary parasitoid hosts after they have egressed from the body of the herbivore hosts or, in the case of aphids, inside the body of the mummified hosts.^6^ Hyperparasitoids can also be classified as ‘obligate’, when they are always hyperparasitoids, and ‘facultative’, when they can develop as a hyperparasitoid in a primary parasitoid or directly in the primary parasitoid's host. Finally, ‘ecto‐hyperparasitoids’ develop on the outside of their hosts (albeit on the inside of the mummy shell or parasitoid silk cocoon) and ‘endo‐hyperparasitoids’ develop inside their primary parasitoid hosts.

Hyperparasitoids have remarkable adaptations that enable them to exploit their parasitoid hosts in the field, and that makes hyperparasitism a very successful strategy. Some primary parasitoids harbor several species of hyperparasitoids in nature (Supporting Information, Table S1 and references therein), and they can locally decimate numbers of parasitoids over several generations.^8^ This role as consumers at the top of insect food chains also means that hyperparasitoids may disrupt biological control of pest insects. Indeed, hyperparasitoids may be one of the greatest threats to the success of parasitoids in biological control programs,^6^, ^9^ particularly in augmentative biological control in greenhouses but also in establishment of parasitoids that are introduced to new areas in classical biological control programs (see Supporting Information, Table S1 for examples). Hyperparasitoids have often been overlooked but their negative impact on biological control may be more common than previously thought. Thus, when biological control programs do not achieve the expected results, it is important to check whether hyperparasitism could be a reason for failure. Careful selection of parasitoid species might prevent problems due to hyperparasitoids in classical biological control, for example by selecting parasitoid species that suffer less from hyperparasitoid‐caused mortality instead of more susceptible species as biological control agents. However, in the case of augmentative biocontrol programs, active management strategies are needed. Such strategies are not currently available and this is in part due to major gaps in our understanding of the biology and ecology of hyperparasitoids.

Earlier studies of hyperparasitoids were largely descriptive and focused on the structure of hyperparasitoid communities associated with primary parasitoid hosts and on the impacts of hyperparasitoids on primary parasitoids in the field, as has been reviewed.^6^ More recently, increasing attention has been paid to life‐history traits of hyperparasitoids that affect hyperparasitoid reproduction and hence fitness, such as development and host‐feeding.^10^, ^11^ Progress has also been made in understanding hyperparasitoid behavior, in particular host‐ and mate‐finding, which are mainly guided by chemical cues (infochemicals).^12^, ^13^ The idea of manipulating hyperparasitoids to enhance biological control was put forward more than two decades ago,^14^, ^15^ but recent progress in chemical ecology makes it possible now to develop truly eco‐friendly pest control, including strategies for managing hyperparasitoids.

Our review covers this topic by (i) detailing the effects of hyperparasitoids on parasitoids in a biological control context, (ii) reviewing the knowledge on the chemical ecology of hyperparasitoids, focusing on infochemicals that may be used to manipulate their behavior, and (iii) developing infochemical‐based management strategies, including a push‐pull strategy to manage hyperparasitoids. We foresee that such strategies may be more successful in greenhouses as compared to the open field because of the advantages of confined spaces and more controlled environmental conditions.

## 2 IMPACT OF HYPERPARASITOIDS ON BIOLOGICAL CONTROL

### 2.1 Occurrence of hyperparasitoids in agricultural crops

Hyperparasitoids are known from various agricultural systems, including greenhouse vegetables, annual field crops, orchard fruits and cultivated forests (Supporting Information, Table S1 and references therein). Although the effects of hyperparasitoids on biological control have not been quantified in most agricultural systems (but see below for exceptions), it is generally thought that the presence of hyperparasitoids decreases the efficacy of biological control. Indeed, in a rare empirical study, using caged miniature fields, the hyperparasitoid *Asaphes suspensus* quickly drove its parasitoid host *Aphidius ervi* to extinction.^8^ Still, populations of the parasitoid were not eliminated in open lucerne fields. Another recent study, using a DNA‐based approach to characterize the aphid‐parasitoid food web in Mediterranean citrus, concluded that hyperparasitoids impede the success of *Binodoxys angelicae* for biological control of pest aphids.^16^ Perhaps surprisingly, hyperparasitoids may also positively affect components of biological control because they can dampen extreme host–parasitoid oscillations and prevent parasitoid extinction by overexploitation of hosts.^6^, ^17^

### 2.2 Effects of hyperparasitoids on their parasitoid hosts

Hyperparasitoids may impact their primary parasitoid hosts in several ways that are relevant to biological control. First, hyperparasitoids have direct negative effects on parasitoids by parasitizing their offspring. Hyperparasitoids can indeed cause substantial mortality to parasitoid species commonly used as biological control agents^6^ (Supporting Information, Table S1 and references therein). Sixteen species of hyperparasitoids were found to attack *Cotesia melanoscela*, which is an important natural enemy of gypsy moth (*Lymantria dispar*) larvae. This hyperparasitoid complex caused approximately 50% mortality of the primary parasitoid. In the Netherlands, at least nine species of hyperparasitoids attack cocoons of *Cotesia glomerata* and *Cotesia rubecula*, two endoparasitoids of *Pieris* caterpillars in wild and cultivated brassicaceous plants. Mortality of these parasitoid cocoons due to hyperparasitoids varies from 9% to more than 80% between years, fields and the two *Cotesia* species. Parasitoids of aphids are also under severe pressure from hyperparasitoids in greenhouse and field crops. Over 80% of parasitoid hosts inside aphid mummies in *Brassica* fields were killed by ten species of hyperparasitoids, while at least nine species of hyperparasitoids caused up to 100% mortality of aphid mummies in sweet pepper greenhouses in two different studies. Failure of aphid pest control with *Aphidius colemani* in sweet pepper and eggplant greenhouses using banker plants (i.e. non‐crop plants providing host resources to parasitoids) was attributed to high rates of hyperparasitism.

A second direct negative effect of hyperparasitoids on their parasitoid hosts is host‐feeding by hyperparasitoids. Host‐feeding occurs when adult females of hymenopteran (hyper)parasitoids consume haemolymph or tissue from the same host species that is also used for oviposition. This behavior can kill the host, depending on the type and extent of host‐feeding.^18^ Host‐feeding has been described for many, but not all, hyperparasitoid species.^6^ However, few studies have quantified the effect of hyperparasitoid host‐feeding on host mortality. To our knowledge, this is only known in one system: 25% of the cocoon stage of *Cotesia melanoscela* was killed by host‐feeding of hyperparasitoids.^19^

Indirect effects of hyperparasitoids on their parasitoid hosts may also influence the efficacy of biological control. In the presence of its hyperparasitoid *Alloxysta victrix*, the aphid parasitoid *Aphidius uzbekistanicus* is less efficient in host exploitation because it attacks fewer hosts and makes more flight attempts compared to control conditions without hyperparasitoids. This suggests that the hyperparasitoid induces dispersal and patch leaving (i.e. the parasitoid leaves a profitable environment in which hosts are present) of the biological control agent.^20^ Aphid hyperparasitoids may also affect the sex ratio of their hosts. Hyperparasitoid presence resulted in lower proportions of emerging females in *Lysiphlebus hirticornis* 21 and *Binodoxys angelicae*.^16^ This phenomenon may be explained by a preference of hyperparasitoids for larger parasitized aphids, which are more likely to contain a female parasitoid. Finally, a study on the parasitoid *Cotesia melitaerum* suggested that generalist hyperparasitoids can mediate apparent competition between *C. melitaerum* and the congeneric *C. glomerata* that attacks another herbivore.^22^ Hence, increases in host abundance of generalist hyperparasitoids in areas close to agricultural fields could indirectly cause increased rates of hyperparasitism on biological control agents and thus reduce the biological control efficacy of pests.

### 2.3 Impact of hyperparasitoids on biological control programs

Native hyperparasitoids may hamper the introduction of exotic biological control agents in classical biological control programs (Table S1 and references therein). For example, after its introduction in southwestern Virginia, *C. rubecula* experienced much higher rates of hyperparasitism than *C. glomerata* that had earlier established in this area. It was hypothesized that hyperparasitism would be a limiting factor in establishing *C. rubecula* as a biological control agent of *Pieris rapae*. Another example is found in biological control of the leek moth *Acrolepiopsis assectella* by the pupal parasitoid *Diadromus pulchellus*. The native facultative hyperparasitoid *Conura albifrons* uses leek moth pupae (including those already parasitized by *D. pulchellus*) as hosts, and its role as a competitor and as an intraguild predator may affect establishment of *D. pulchellus*. However, the presence of native hyperparasitoids does not always preclude the success of classical biological control programs, as demonstrated in biological control of the cassava mealybug in Africa.^6^ Although the parasitoid *Apoanagyrus lopezi* is attacked by at least ten different indigenous hyperparasitoids, sometimes at high hyperparasitism rates, its introduction is still considered economically successful. Indeed, theoretical models have shown that hyperparasitoids may dampen extreme host‐parasitoid oscillations and can thereby contribute to the stability of multitrophic systems. Increased stability would allow parasitoids to persist in the crop and prevent pest outbreaks that may occur in the absence of parasitoids, thus retaining the efficacy of biological control.^23^ However, such situations may be rare and, in most cases, invading hyperparasitoids are more likely to disrupt stable host–parasitoid interactions. Climate change may increase the frequency and magnitude of disruption of biological control by hyperparasitoids in the near future due to biological invasions or shifts in phenology.^24^

The examples in this section not only show substantial mortality of parasitoids due to hyperparasitoids in agricultural systems, but also illustrate that hyperparasitoid communities are often highly diverse, with multiple hyperparasitoid species attacking one primary parasitoid. Within systems, the species composition of hyperparasitoids may further vary in space and over the season. In a study on *C. glomerata* cocoons attached to leaves at the bottom of the plant or in the canopy, it was shown that wingless species such as *Gelis proximus* were predominant in the vegetation close to the ground whereas winged species such as *Acrolyta nens* were more abundant in the canopy. This demonstrates that community composition of hyperparasitoids can greatly vary even at small spatial scales.^25^ Temporal changes in hyperparasitoid communities were recorded in a monthly survey of aphid hyperparasitoids in organic sweet pepper greenhouses, where *Dendrocerus aphidum* was more abundant before summer and *Asaphes vulgaris* became more abundant in late summer (Table S1). This diversity and variation presents a challenge in managing hyperparasitoids in biological pest control because a successful strategy against one species may not be effective against another species. Conversely, most of the hyperparasitoid species mentioned above have a broad host‐range and many of them occur across different combinations of crops and pests, such as the suite of aphid hyperparasitoids (attacking mainly *Aphidius* and *Aphelinus* parasitoids) or the hyperparasitoid communities attacking *Cotesia* cocoons. These systems could serve as case studies for the development of hyperparasitoid management strategies.

In terms of their impact on the crop, hyperparasitoids are in fact pest insects, being natural enemies of the natural enemies of herbivorous pest insects. In developing sustainable management strategies for hyperparasitoids, knowledge on herbivore pest management strategies may therefore be applied. In addition, as in strategies to manage crop pests, the impact of hyperparasitoid management strategies on non‐target organisms must be minimized. Specifically, such a strategy should not interfere negatively with biological control agents of the target herbivore pest. Because hyperparasitoids are biologically very similar to their parasitoid hosts, this is a main challenge in developing management strategies. For example, if a hyperparasitoid trap was available, it should not also trap primary parasitoids or other natural enemies. It is therefore important to consider the specificity of the lure to bait such a trap (and to consider aspects of trap design or implementation) to minimize the impact on beneficial insects. Because infochemicals are very important in mediating the behavior of many insects,^26^ and because such chemical cues usually have species‐specific effects, we propose that studying the chemical interactions between hyperparasitoids, their parasitoid hosts, herbivores and plants is an essential first step.

## 3 INFOCHEMICALS THAT CAN BE USED TO MANIPULATE HYPERPARASITOID BEHAVIOR

**Figure 1:**
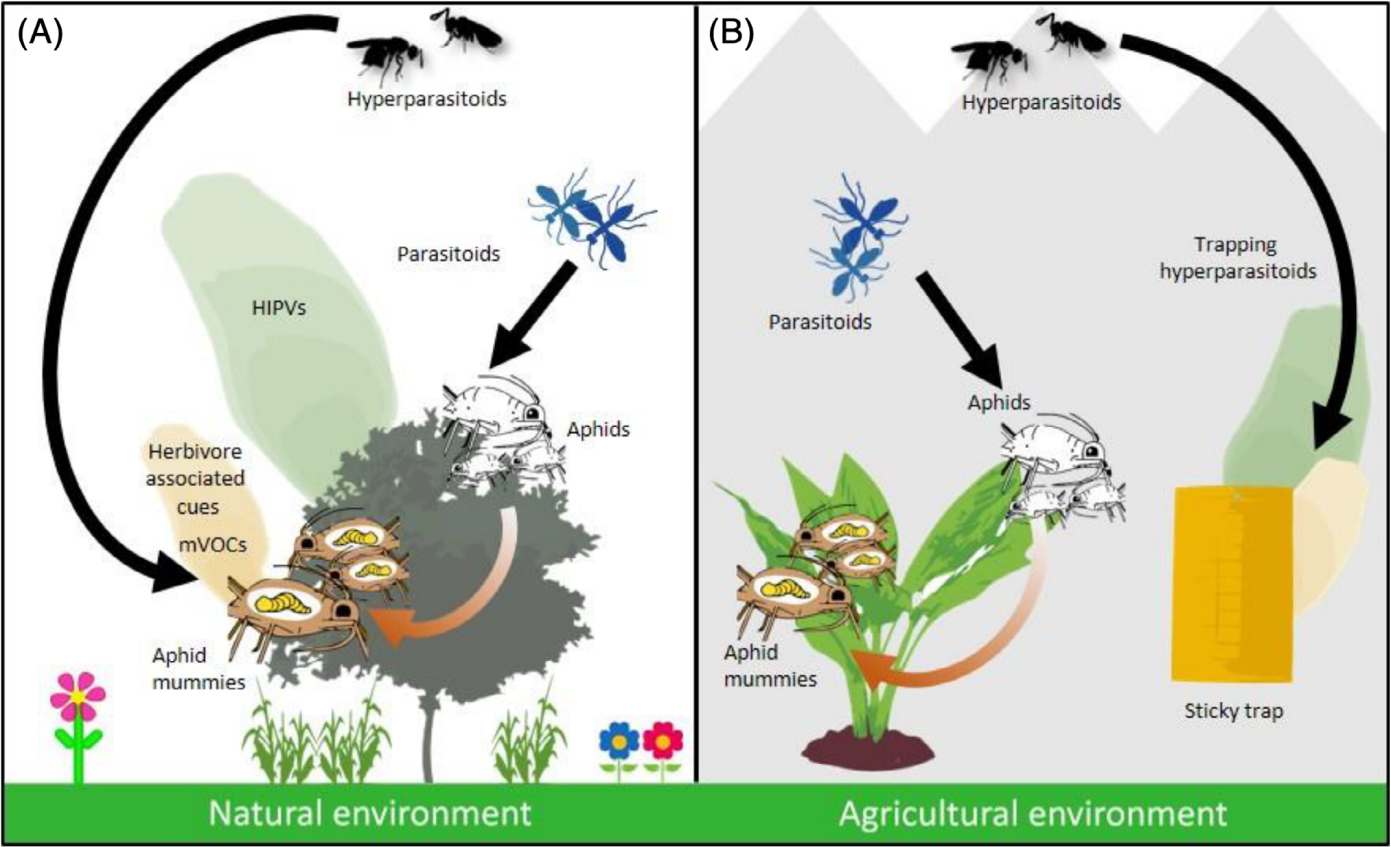
Infochemical‐based searching behavior of hyperparasitoids. (A) In the natural environment, hyperparasitoids find their parasitoid hosts by exploiting plant‐derived chemical cues (HIPVs) and cues associated with the parasitized herbivores. (B) In the agricultural environment, the same infochemicals could be used in management strategies to divert hyperparasitoids away from parasitized herbivores and lure them towards point‐source attraction devices such as sticky traps. HIPVs, herbivore‐induced plant volatiles; mVOCs, microbial volatile organic compounds.

Infochemicals are used by insects in mate, host or food location and the (blends of) chemical compounds that guide these behaviors could be exploited to manipulate hyperparasitoid behavior. Infochemicals can originate from various sources in the hyperparasitoid's environment and may mediate interactions between hyperparasitoids and other organisms (Fig. 1). Infochemicals can function as inter‐specific signals (e.g. plant volatiles or cues associated with parasitized herbivores) or act intra‐specifically (e.g. pheromones) (Table 1). It is also possible that hyperparasitoids respond to signals that are used in communication between or within other species, i.e. they ‘eavesdrop’. In this section we list the infochemicals that can be used to manipulate hyperparasitoid behavior, whereas in Section 4 we discuss how to employ such chemical cues in possible management strategies.

**Table 1:** Hyperparasitioid species known to respond to infochemicals. Species are grouped according to three main categories of infochemicals: plant‐derived cues, herbivore‐derived cues or pheromones

Hyperparasitoid species	Infochemical source	Effect on hyperparasitoid	Chemical characterization	Reference
	**Plant‐derived cues**			
*Alloxysta pleuralis*	Plants extracts	Attraction to foliage extracts of a range of plant species (including the host plant pigeon pea *Cajanus cajan*)	Active compounds soluble in water	31, 32
*Alloxysta victrix*	Plant‐herbivore complex	Attraction to volatiles from a combination of oat leaves (*Avena sativa*) and aphids	Active compounds: (*E*)‐β‐farnesene and linalool	33
*Dendrocerus carpenteri*	Plant‐herbivore complex	Attraction to volatiles from a combination of oat leaves (*Avena sativa*) and aphids	NA	33
*Eunera augarus*	Plant volatiles	Preference for volatiles of coniferous plants (*Pinus sylvestris*) over non coniferous plants (*Betula pendula*)	NA	13
*Lysibia nana*	HIPVs	Attraction towards HIPVs emitted by cabbage plants (*Brassica oleracea*) induced by parasitized caterpillars over unparasitized caterpillars	Parasitization induced changes in the quantitative composition of the blend of HIPVs (*E*)‐DMNT was present in higher concentrations in plants damaged by *Cotesia glomerata* –parasitized caterpillars	12, 28, 29
*Pteromalus semotus*	HIPVs	Attraction towards HIPVs emitted by cabbage plants (*Brassica oleracea*) induced by parasitized caterpillars Preferences for the cultivar Christmas Drumhead over Badger Shipper	NA	30
	**Herbivore‐derived cues**			
*Alloxysta victrix*	Parasitized aphids	Arrestment response leading to a significantly longer residence time compared with unparasitized aphids	Solubility in hexane Possible involvement of cuticular hydrocarbons	15
	Aphids	Attraction to the aphid‐alarm pheromone	Active compound: (*E*)‐ β‐farnesene	33
	Honeydew	Arrestment response leading to increased residence times in substrates contaminated with the aphid honeydew No response to scale honeydew No discrimination between honeydew from parasitized and unparasitized aphids	Active compounds soluble in water	37, 38
*Baryscapus galactopus*	Caterpillar body odors	Attraction to volatiles released by parasitized caterpillars over unparasitized caterpillars	Parasitization changes the quantitative composition of the blend of body odors 2,3‐butanedione was present in higher concentrations in the headspace of parasitized caterpillars	36
*Dendrocerus carpenteri*.	Honeydew	Arrestment response leading to increased residence times in substrates contaminated with the aphid honeydew No response to scale honeydew No discrimination between honeydew from parasitized and unparasitized aphids	Active compounds soluble in water	37, 38
*Dendrocerus carpenteri*	Aphid mummies	Attraction to volatiles emitted by aphid mummies with or without healthy aphids present	Hexane extracts contained 11 compounds (long‐chain alkanes, aldehydes and alcohols C25–C33) which were active as mixture but not as single compounds	33
*Phaenoglyphis villosa*	Honeydew	Arrestment response leading to increased residence times in substrates contaminated with aphid honeydew	Active compounds soluble in water	37
*Syrphophagus aphidivorus*	Honeydew	Arrestment response leading to increased residence times in substrates contaminated with the aphid honeydew No response to scale honeydew No discrimination between honeydew from parasitized and unparasitized aphids	Active compounds soluble in water	38
	**Pheromones**			
*Alloxysta victrix*	Females and males	The chemical cue attracts males (volatile sex pheromone) and repels females (putative spacing pheromone)	Active compound: MHO or sulcatone	14
*Dendrocerus carpenteri*	Females	Volatile sex pheromone attracts conspecific males. Female attractiveness depends on age and mating status	NA	42
		Marking pheromone applied on the mummy shell after oviposition prevents superparasitism	NA	43
		Marking pheromone applied on the substrate reduces repeated exploration of previously visited patches	Juvenile hormone	44

HIPVs, herbivore‐induced plant volatiles; (*E*)‐DMNT, (*E*)‐4,8‐dimethylnona‐1,3,7‐triene; MHO, 6‐methyl‐5‐hepten‐2‐one; NA, no information available.

### 3.1 Herbivore‐induced plant volatiles and other plant‐derived cues

Although herbivore‐induced plant volatiles (HIPVs) are important for many parasitoids of herbivorous hosts,^27^ only a few species of hyperparasitoids are known to respond to HIPVs. *Lysibia nana*, a specialized ecto‐hyperparasitoid of hosts within the genus *Cotesia*, prefers HIPVs emitted by cabbage plants attacked by parasitized caterpillars over plant volatiles emitted in response to unparasitized caterpillars.^28^ The chemical composition of the HIPV blend changes according to the parasitism status of the attacking herbivore, allowing hyperparasitoids to locate their hosts. Interestingly, HIPV attraction of *L. nana* occurs regardless of the host caterpillars in which *C. glomerata* parasitoid larvae develop (*Pieris brassicae* or *P. rapae*), showing that the parasitoid signature overrules herbivore identity in this system.^12^, ^29^ *Lysibia nana* and also *Pteromalus semotus* are attracted to HIPVs induced by parasitized caterpillars on different cabbage cultivars and both hyperparasitoids preferred cultivars that also are more attractive to their primary parasitoid host *C. glomerata*.^30^

While HIPVs emitted in response to parasitized herbivores may offer detectable and reliable cues for foraging hyperparasitoids, as they do for primary parasitoids,^27^ there is no evidence yet that they are used by hyperparasitoids associated with aphids. Several studies have investigated aphid hyperparasitoid responses to plant volatiles, but the role of plants in mediating the attraction of this group of hyperparasitoids remains unclear. *Eunera augarus*, an ecto‐hyperparasitoid specialized on aphids feeding on coniferous plants, discriminates between odors of coniferous (i.e. *Pinus sylvestris*) versus non‐coniferous plants (i.e. *Betula pendula*), irrespective of the presence of its mummy host.^13^ The aphid endo‐hyperparasitoid *Alloxysta pleuralis* responds to foliar extracts of plants,^31^, ^32^ while the congeneric *A. victrix* and the generalist ecto‐hyperparasitoid *Dendrocerus carpenteri* are attracted to a combination of oat leaves and aphids.^33^ In contrast, there was no attraction of four unrelated species of hyperparasitoids (*D. carpenteri*, *Asaphes suspensus*, *A. victrix* and *Syrphophagus aphidivorus*) towards volatiles from the potato plant–host complex.^34^ The lack of a clear role for HIPVs in the host‐searching behavior of aphid hyperparasitoids could be due to a paucity of studies after the first discovery of this phenomenon in the caterpillar‐associated hyperparasitoid *L. nana*. Using HIPVs may be particularly adaptive for aphid endo‐hyperparasitoid species that attack parasitized aphids that actively feed on plants. This advantage may be less obvious for ecto‐hyperparasitoids because their mummy hosts no longer feed on the plant and such hyperparasitoids can usually attack a wide range of aphid‐parasitoid combinations.^6^ An indication that HIPV composition may be influenced by the parasitism status of aphids comes from recent work showing that a jasmonic acid‐responsive gene is differentially expressed after infestation with parasitized versus healthy aphids.^35^

### 3.2 Herbivore‐derived cues

Evidence that hyperparasitoids exploit herbivore‐derived cues when foraging for hosts is available for caterpillar‐ as well as aphid‐associated hyperparasitoids. For example, the endo‐hyperparasitoid *Baryscapus galactopus* uses body odors of the herbivore to discriminate between unparasitized caterpillars and those carrying parasitoid host larvae.^36^ The endo‐hyperparasitoid *A. victrix* responds strongly to hexane extracts of parasitized aphids, while unparasitized aphids elicit only a weak response. This suggests that apolar compounds, such as cuticular hydrocarbons, may be involved in hyperparasitoid recognition of *Myzus persicae* aphids carrying *Aphidius colemani* parasitoid larvae.^15^

Honeydew, excreted by aphids, may also emit infochemicals that can be used by aphid‐associated hyperparasitoids. Honeydew is a valuable food source that can extend the longevity of hyperparasitoid species in the genera *Asaphes* and *Dendrocerus*,^11^ but it may also indicate the presence of hosts to hyperparasitoids. Indeed, aphid hyperparasitoids from different families (Megaspilidae, Alloxystidae, Encyrtidae) respond to substrates contaminated with aphid honeydew by investigating the surrounding areas by antennal drumming, while flight is suppressed (i.e. arrestment behavior).^15^, ^37^ Infochemicals from honeydew appear to be somewhat specific because three hyperparasitoid species did not respond to honeydew produced by scale insects, which are non‐hosts for the primary parasitoids.^38^ This ability to discriminate between aphid and non‐aphid honeydew may allow hyperparasitoids to focus on searching in areas where aphid parasitoids are probably present. Nevertheless, hyperparasitoids did not discriminate between honeydew from parasitized and unparasitized aphids,^38^ even though parasitism may change the composition of honeydew, at least in terms of amino acid concentrations and ratios,^39^ and this could reveal important information about the presence of the primary parasitoid to hyperparasitoids. Furthermore, attraction of hyperparasitoids to honeydew over longer distances (i.e. during in‐flight foraging) has not yet been shown. The importance of honeydew infochemicals in managing hyperparasitoids is therefore likely to be limited compared to more specific signals such as cuticular compounds or HIPVs, the latter having the additional advantage of being attractive over larger distances.

### 3.3 Pheromones

Pheromones are an interesting group of infochemicals employed in management of hyperparasitoids. Pheromones mediate intraspecific communication and are important in insects for mate finding and recognition, for aggregation of individuals of both sexes, and for marking previously visited patches or parasitized hosts to prevent superparasitism. An extensive body of literature on pheromones exists for herbivorous insects,^40^, ^41^ while research on the effects of pheromones on parasitoids and hyperparasitoids has lagged behind. The limited information available for hyperparasitoids illustrates the complexity of intraspecific communication, at least in some species. For example, three different pheromones have been described so far for the aphid ecto‐hyperparasitoid *Dendrocerus carpenteri*: (i) a volatile sex pheromone released by females that attracts males,^42^ (ii) an external marking pheromone deposited on mummies after oviposition that prevents superparasitism,^43^ and (iii) external marks applied on the substrate that reduce repeated exploration of previously visited patches (i.e. areas with hosts).^44^ The chemical cues used to mark parasitized aphids appear to be composed of two externally perceivable chemical markers that are deposited on mummy shells: one marker is short‐lived but strongly active whereas the other marker is more persistent but moderately active.^43^ Furthermore, behavioral assays suggest that the infochemicals left on patches with hosts are different from marks left on patches where no hosts have been found.^44^ The aphid hyperparasitoid *Alloxysta victrix* produces a volatile sex pheromone whose major compound, 6‐methyl‐5‐hepten‐2‐one (MHO, also known as sulcatone), has been suggested to act as a sex pheromone.^14^ Surprisingly, there is no evidence that MHO plays a role in intraspecific communication in the closely related species *A. brevis*.^45^ (*E*)‐β‐farnesene, which is an aphid alarm‐pheromone, attracts the aphid hyperparasitoid *A. victrix*, albeit at low response rates,^33^ suggesting that hyperparasitoids may indeed eavesdrop on communication between other organisms.

### 3.4 Microbial volatiles

Some microbial volatile organic compounds (mVOCs) can be insect infochemicals. This discovery has recently opened up a new field in chemical ecology and mVOCs are potentially very important for communication between higher organisms, such as plants and insects.^46^ In fact, manipulating insect–microbe chemical communication could be a novel approach to control insect pests in agriculture.^47^ Unfortunately, virtually nothing is known on the role of microbial volatiles in the chemical ecology of fourth trophic level organisms. Future research efforts should be made to elucidate whether mVOCs are reliable signals for hyperparasitoids during mate and host finding, and whether they may be exploited in infochemical‐based management strategies.

## 4 DEVELOPING PUSH‐PULL AND OTHER INFOCHEMICAL‐BASED STRATEGIES TO MANAGE HYPERPARASITOIDS

### 4.1 Push‐pull strategies in pest control

Infochemicals have been used successfully in pest control to monitor insect pest populations, to remove pests from the environment (mass trapping), or to interfere with their behavior to reduce damage to the crop.^48^ Most of these infochemical‐based pest management strategies rely on pheromones of the targeted species. Plant volatiles have been implemented most successfully in push‐pull strategies to manipulate the behavior of pests and/or their natural enemies.^49^, ^50^

A push‐pull strategy consists of a two‐component approach in which infochemicals are combined with each other to alter the behavior of the target organisms (either the pests or their natural enemies) in order to influence their abundance and distribution in the crop.^49^ When the target organism is a pest insect, repellents or deterrents to ‘push’ them away from the crop are combined with attractant or stimulant cues to ‘pull’ them into other areas such as a neighboring trap crop or into baited traps. In the case of parasitoids, the goal is to combine chemical cues to push them out of the area surrounding the crop and to pull them into the focal crop. Push‐pull implementation has in some cases achieved promising results, for example in management of stemborers in maize and sorghum in sub‐Saharan Africa.^51^ Molasses grass *Melinis minutiflora* (push component) emits compounds such as (*E*)‐β‐ocimene and (*E*)‐4,8‐dimethyl‐1,3,7‐nonatriene that are repellent in pest oviposition assays.^50^ Napier grass *Pennisetum purpureum* (pull component) is used as the trap crop in which stemborers oviposit heavily despite the strong mortality experienced by the developing larvae.^52^ An advantage of a push‐pull strategy, compared with strategies based on a single infochemical, is to exploit the combined effects of the attractive and repellent components, which often act in synergy to influence insect behavior.^49^ In fact, either push or pull component may not be (sufficiently) effective when acting alone because their effects may be not strong enough to modify the distribution/abundance of the targeted insects.

### 4.2 Push‐pull strategies to manipulate hyperparasitoids

**Figure 2:**
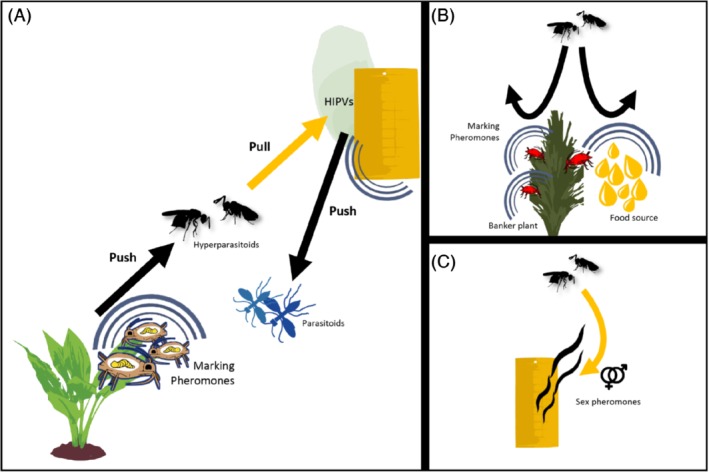
Infochemical‐based strategies to manage hyperparasitoids in agricultural environments. (A) Possible push‐pull approach where marking pheromones are used to push hyperparasitoids away from parasitized herbivores and HIPVs (derived from plants induced by parasitized herbivores) are used to pull hyperparasitoids towards a trapping device. HIPVs derived from plants induced by parasitized herbivores also push parasitoids away from the trap, thus minimizing the removal of biological control agents from the agricultural environment. (B) Marking pheromones can be used to limit the accessibility of resources (e.g. sugars or parasitized aphids present in banker plants) to hyperparasitoids. (C) Sex pheromones can be used to monitor hyperparasitoid presence for early detection in the agricultural environment.

We suggest that a push‐pull strategy could also be an interesting approach for hyperparasitoid management because a combination of infochemicals may be selected to effectively remove hyperparasitoids from the agricultural environment, while minimizing the impact on beneficial insects. Different infochemicals may be combined to push hyperparasitoids away from their parasitoid hosts and to pull them into traps, thus releasing primary parasitoids from hyperparasitoid pressure. Alternatively, the same infochemical (blend) may be used to simultaneously pull hyperparasitoids into a trap and to push primary parasitoids away from the trap, thus minimizing the removal of beneficial natural enemies from the crop (Fig. 2). This alternative push‐pull strategy is similar to conventional mass trapping, with the difference that the used infochemicals have the dual function of attracting hyperparasitoids and repelling primary parasitoids so that only hyperparasitoids would be caught in traps and removed from agricultural settings.

Hyperparasitoid pheromones may be particularly suitable as push components. For example, external host‐marking pheromones, used by hyperparasitoids to prevent superparasitism or to mark previously explored areas, would interfere with hyperparasitoid foraging behavior, effectively disguising parasitized hosts as already exploited resources. As such, a hyperparasitoid marking pheromone could be used to protect primary parasitoids (i.e. the biological control agents) from hyperparasitism upon introduction into the greenhouse. This may be important for parasitoids attacked by species of *Dendrocerus* and *Asaphes* that attack aphid mummies until approximately a day before primary parasitoids emerge.^11^, ^53^ Marking pheromones act at short range so a possible strategy could be to directly apply the pheromone to the release devices with the biological control agents (e.g. the containers with mummies could be coated with the pheromone), whereas the use of dispensers may be less effective given the low volatility of these compounds. Spraying pheromones onto the crop may be effective but requires assays first to verify if this leads to interactions between the pheromones and the plants. Infochemicals that act over a longer range than currently identified aphid hyperparasitoid marking pheromones would be even more suitable as a first line of defense against hyperparasitoid attack. However, to date, no other sources of infochemicals that repel hyperparasitoids have been found, likely due to the fact that hyperparasitoids are understudied as pest insects. The novel (and largely unknown) field of insect–microbe communication may provide opportunities to explore whether there are mVOCs that specifically repel hyperparasitoids, and not their primary parasitoid hosts. If such compounds are discovered, they could be synthetized and implemented in an infochemical‐based management strategy. Alternatively, mVOCs may be produced by the repellent microbes directly. Microbes could be grown in containers with media suitable for bacterial and/or fungal growth and placed in the crop. We think this approach would be more successful than spraying microbes directly onto plants or coating seeds with microbes because of possible interactive effects of bacterial/fungal cells with the crop.

Pheromones have been successfully applied as pull components in monitoring and mass trapping of herbivorous insects.^41^ For example, female cerambycid beetles produce a sex pheromone that attracts conspecific males and additionally acts as an aggregation pheromone (i.e. by also attracting conspecific females) thus recruiting conspecifics of both sexes.^40^ The latter property makes such pheromones particularly useful because all individuals in the pest population are targeted. Whereas no aggregation pheromones have been identified in hyperparasitoids, evidence of sexual communication is available for species in the genera *Dendrocerus* and *Alloxysta*, where males are attracted to conspecific females.^14^, ^42^ In mass trapping, removing only males from the crop would be a limitation of using sex pheromones because the female hyperparasitoids that kill the primary parasitoids by laying eggs in their host would not be targeted. Nonetheless, such infochemicals could still be used in monitoring traps (Fig. 2 and below). The species‐specificity of sex pheromones can be an advantage because this may reduce the impact on behavior of beneficial insects, but it may also be a disadvantage because multiple pull stimuli would be needed when the biocontrol agent is attacked by a complex of hyperparasitoids. In the case of MHO, its function as a sex pheromone in *Alloxysta victrix* is apparently indeed highly species‐specific because the congeneric *A. brevis* is not attracted.^14^, ^45^ However, MHO induces dispersal in the aphid parasitoid *Aphidius uzbekistanicus*, potentially interfering with its foraging efficiency.^20^ Due to this negative effect on the beneficial insects, using MHO for mating disruption of *Alloxysta victrix* may not be advised because a high number of lures (and thus high concentration of the chemical compound) would likely be needed to interfere with the pheromonal communication of the targeted hyperparasitoids.

HIPVs are also of potential interest for push‐pull strategies because they may simultaneously affect both hyperparasitoids and their parasitoid hosts. Cabbage plants emit HIPVs in response to feeding by parasitized caterpillars that attract hyperparasitoids^28^ but repel primary parasitoids.^54^ Repellence of the latter by these HIPVs is thought to reduce intra‐specific competition and to improve the efficiency of parasitoids as they do not waste time exploring a plant containing already exploited resources. Because HIPVs emitted in response to parasitized pests selectively attract caterpillar‐associated hyperparasitoids, their implementation in biological pest control programs is promising. Furthermore, traps baited with HIPVs are expected to mainly attract hyperparasitoid females and this should lead to an effective decline of the hyperparasitoid population. As described above, hyperparasitoids can also exploit cues from parasitized pest insects but because these cues (body odors of caterpillars and cuticular hydrocarbons of aphids) are perceived at short range, their value as a pull component is limited compared to HIPVs, which are airborne signals that generally act over larger distances.

To summarize, to control hyperparasitoids with push‐pull, perhaps the most effective approach would be to combine the most promising stimuli discussed above (HIPVs as pull, marking pheromones as push) to achieve multiple beneficial effects, such as (i) protection of developing parasitoids, which is especially important when the stage of the biological control agent that is released in the crop is also the suitable host stage for hyperparasitoids, (ii) attraction of adult hyperparasitoids towards the trap, and (iii) repellence of primary parasitoids away from the trap (Fig. 2(A)).

### 4.3 Other infochemical‐based strategies to manage hyperparasitoids

Disrupting hyperparasitoid behavior with repellent/deterrent infochemicals could also be used in strategies to support primary parasitoids without also supporting their hyperparasitoid natural enemies, e.g. in a conservation biological control context. For example, melibiose is a sugar commonly found in floral nectar, which benefits primary aphid parasitoids (*Aphidius colemani* and *A. matricariae*) more than the hyperparasitoid *Dendrocerus aphidum*.^55^ Using repellent/deterrent infochemicals in crop areas where sugar sources are provided could further limit the accessibility of these resources to hyperparasitoids. A similar approach could be used to optimize the use of banker plants in greenhouses: these are non‐crop plants (often cereals) infested with aphid species that do not attack the crop, yet the aphids are suitable hosts for parasitoid species, which can thus increase their abundance in the greenhouse. Banker plants thus enhance the complexity of simplified confined agro‐ecosystems and represent a reservoir of alternative hosts for primary parasitoids but may at the same time provide a jackpot to hyperparasitoids (Fig. 2(B)).^56^ The same risk may exist when companion plants are used to provide alternative food or hosts in conservation biological control in the open field.^57^ Thus, chemical ecology could help to tailor these biocontrol strategies to selectively benefit the biological control agent, provided infochemicals can be found that influence the behavior of primary and hyperparasitoids differentially.

Knowledge of the chemical ecology of hyperparasitoids can also be applied in other ways. For example, hyperparasitoids could be detected at an early stage by using monitoring traps baited with sex pheromones or other attractive infochemicals. Early detection could be particularly useful in those situations where hyperparasitoids cannot be effectively managed. In these cases, to prevent disruption of biological control, a possible solution could be to switch from regular releases of primary parasitoids towards releasing predatory natural enemies. In this situation, monitoring traps for hyperparasitoids allow the timing of the release of the alternative biocontrol agent immune to hyperparasitoids to be optimized (Fig. 2(C)).

## 5 CONCLUSION AND FUTURE RESEARCH

The few studies on the use of infochemicals by hyperparasitoids have highlighted that these top carnivores respond to a variety of plant‐ and insect‐derived cues. However, to exploit infochemicals in hyperparasitoid management strategies to enhance biological pest control, a more thorough understanding of their chemical ecology is needed. This will be challenging given the diversity of hyperparasitoid communities that may attack the parasitoid species used for biological control. Up to now, little is known about infochemicals that elicit long‐range repellence and that could be employed to ‘push’ hyperparasitoids away from their parasitoid hosts. mVOCs represent an untapped potential source of infochemicals for dealing with hyperparasitoids and further research is needed to unravel their function, especially to clarify whether mVOCs could act as long‐range repellents. Current information on hyperparasitoid chemical ecology is scarce and scattered over different biological systems, precluding generalizations. In particular, we do not yet understand how widely HIPVs are used by hyperparasitoids and consequently what the true potential of such infochemicals as ‘pull’ components is. There is evidence that caterpillar‐associated hyperparasitoids can discriminate between HIPVs induced by parasitized and unparasitized herbivores, but whether aphid‐associated hyperparasitoids are capable of doing so remains to be shown.

### 5.1 A research agenda to identify infochemicals to manage hyperparasitoids

Future research efforts should identify infochemicals that mediate the behavior of hyperparasitoids, particularly of aphid‐associated hyperparasitoids because they are often responsible for disrupting biological control in greenhouses (Table S1). A possible research approach could consist of the following steps: (i) chemical analyses to identify the chemical composition of the biological source known to be attractive or repellent for hyperparasitoids, (ii) electroantennogram studies and/or behavioral assays with synthetic chemical compounds to select a subset of candidate infochemicals present in the complete blends, and (iii) greenhouse (or field) tests with selected candidate compounds, including evaluating the impact on non‐targets, particularly natural enemies of the pest insect. Key compounds that have a role as hyperparasitoid pheromones have been identified for a few aphid‐associated species and these chemicals could already be tested in a greenhouse setting to develop attractive lures (phase 3). Unfortunately, attractive compounds such as the commercially available MHO also induce the dispersal of third trophic level parasitoids, warranting special attention to evaluate whether the benefits of decreased hyperparasitoid populations by using MHO‐based lures outweigh the costs of a reduction in parasitoid efficiency.

An important next step in designing an effective infochemical‐based management strategy concerns the optimal concentration of active compounds. This is a challenging step because optimal concentrations are not known for many carnivores, including hyperparasitoids, and may differ between the hyperparasitoid species present in the crop. Studies on third trophic level parasitoids and predatory mites suggest that attraction towards infochemicals is likely to be maximized at intermediate concentrations due to a dose–response relationship that is hump‐shaped.^58^, ^59^ Thus, further studies should investigate hyperparasitoid responses to different doses of candidate chemical compound(s) to unravel which concentrations are the most effective in attracting hyperparasitoids. At the same time, varying the concentration of infochemicals presents an opportunity to minimize the impact on beneficial natural enemies because optimal concentrations are likely to be species‐specific. The effectiveness of a lure is also determined by the medium from which the lure is released and by the density of lures installed in the crop, and these aspects need to be optimized to maximize the effectiveness of the management strategy in a cost‐effective way. Furthermore, because applications must be easy to use for growers, passive systems (i.e. rubber dispensers or coated traps) are probably better than active systems (i.e. air streams in greenhouses). Slow release dispensers to deliver optimal rates of infochemicals tailored to each hyperparasitoid species may be the best solution in such challenging situations because they can deliver constant rates over long periods of time.

Finally, trap design and placement are key aspects for effective hyperparasitoid management. Because the goal is to remove hyperparasitoids, point‐source attraction devices such as sticky traps or delta traps baited with the attractive lure can be employed. These traps are relatively cheap and widely available on the market. The placement of the trap likely depends on the specific cropping system, and height may be varied throughout the season due to plant growth. Trap color should be considered because hyperparasitoids may respond to different colors to their primary parasitoid hosts. For example, the aphid hyperparasitoid *Pachyneuron aphidis* is sensitive to yellow light, unlike its host *Aphidius gifuensis*,^60^, ^61^ suggesting that yellow sticky traps would be a good choice in this case. The number of hyperparasitoids present in/on traps could be used to assess trap efficacy and inspections should also evaluate how many beneficial insects are removed from the agricultural environment. During development – and ideally also during commercial trap use – assessments of trap efficacy should also include functional changes with respect to biological control of the pest insect (i.e. whether there is an effective drop in hyperparasitism rates).

### 5.2 Targeting hyperparasitoids with transgenic plants or with plants primed for defenses

An alternative approach to the use of synthetic infochemicals to manage hyperparasitoids is the use of transgenic plants that emit such infochemicals.^62^ For example, wheat plants exist that overexpress (*E*)‐β‐farnesene, the alarm pheromone for many pest aphid species. However, using these plants did not lead to the expected level of biological pest control, likely because plants without pest insects continuously emit this infochemical, which may disrupt the attraction of primary parasitoids. When parasitoids are attracted to plants without herbivore hosts, they may become habituated to the infochemical, leading to a reduction or even lack of response to the overexpressed infochemical.^62^ How hyperparasitoids would respond to such transgenic plants under laboratory and field conditions remains to be investigated. A disadvantage of the continuous emission of volatile compounds from uninfested plants are the metabolic costs involved. Instead of using their resources for growth, plants may ‘waste’ resources to produce chemical compounds, even when hyperparasitoids are not present in the environment. To overcome these problems, plant ‘priming’ (a physiological state in which a plant is conditioned for faster and stronger defense activation) may be a more useful strategy because defenses are not active continuously.^63^ Plant priming occurs when uninfested plants respond to signals indicating herbivore presence in the environment (i.e. HIPVs from neighboring infested plants) and it allows plants to prepare their defenses when herbivory occurs. So far, knowledge of the effects of plant priming on parasitoids is scarce and nothing is known for hyperparasitoids, so it is challenging to predict if and how this strategy could be used to manage hyperparasitoids.

### 5.3 Challenges and perspectives for infochemical‐based hyperparasitoid management

We suggest that developing hyperparasitoid management strategies in confined environments such as greenhouses provide the best opportunity, both from an economical and biological perspective. In confined environments, hyperparasitoids build up their populations faster than in the field, potentially leading to the extinction of the pest's natural enemies.^8^ Developing efficient biological control methods is therefore especially needed in protected agriculture considering that fewer new insecticides are becoming available on the market and pesticide applications may be particularly harmful for workers in confined environments.^64^ This makes the economic benefits of developing trapping systems for hyperparasitoids greater in protected agriculture than in open fields. Moreover, environmental conditions are much more variable in open fields, which may hamper successful implementation of infochemical‐based strategies. In fact, an extensive body of literature exists on attraction of parasitoids and other carnivores to infochemicals in the laboratory, yet implementation of infochemical‐based tactics to manipulate parasitoid behavior is limited, with failures possibly due to the complexity of field cropping systems.^62^, ^65^ In greenhouses, the application of infochemicals has not yet been considered in a biological control context because the focus has been on third trophic level parasitoids that are released and retained in the closed system so there is limited need to lure them into crops from surrounding areas.^66^ However, the emerging pattern that hyperparasitoids can disrupt commercial biological control by parasitoids opens new scenarios for implementation of infochemicals in greenhouses. It is difficult to predict the actual feasibility and the likelihood of implementation of push‐pull strategies to manage hyperparasitoids. So far, no studies attempted to achieve this goal and certainly carrying out semi‐field experiments with synthetic compounds is a crucial step towards understanding the real potential of this infochemical‐based approach. Currently, no other effective strategies are available for growers when hyperparasitoids disrupt biological control. In fact, under such circumstances, the only pesticide‐free solution may be to switch from parasitoids to other biological control agents that are less susceptible or immune to hyperparasitoids (i.e. predators). Considering the need to find strategies that can replace pesticides in protected agriculture, we see a need to invest in developing a push‐pull approach, which has proven to be very successful in pest management in other cropping systems.^49^

Shifting from pesticide application to biological control requires a better understanding of food web functioning, as four‐trophic level systems commonly occur, even in simple agroecosystems such as greenhouses. Research on chemical ecology should therefore be integrated with community ecology to evaluate the impact of hyperparasitoid management at each trophic level in the food web. In this sense, infochemical‐based tactics that rely on HIPVs provide more challenges compared with hyperparasitoid pheromones. The latter chemical signals are generally highly specific to a single species and sex, whereas plant volatile compounds are known to affect organisms of different trophic levels, including neighboring plants, pests and natural enemies.^27^, ^67^, ^68^ Moreover, HIPVs usually consist of complex blends of volatiles, of which one or more compounds play a role in mediating insect behavior, often in specific ratios.^69^, ^70^ Regardless of whether plant‐derived or insect‐derived chemical cues are used, it is important that the compound selectively targets hyperparasitoids without interfering with the activity of primary parasitoids. To conclude, managing hyperparasitoids in greenhouse crop cultivation using infochemicals is a promising approach but many research efforts are still needed to develop effective (push‐pull) strategies.

## Supporting information


**Table S1** Examples of crops in different agricultural systems in which hyperparasitoids attack parasitoids used in the biological control of pest insects.
