# Supplementary material for: Exploiting chemical ecology to manage hyperparasitoids in biological control of arthropod pests
Source: Pest Manag Sci. 2019 Dec 4;76(2):432–43. doi: 10.1002/ps.5679 (PMC7004005; doi:10.1002/ps.5679)
Supplement: Supplementary file 1 — Table S1 Examples of crops in different agricultural systems in which hyperparasitoids attack parasitoids used in the biological control of pest insects. [file PS-76-432-s001.docx]

Table S1. Examples of crops in different agricultural systems in which hyperparasitoids attack parasitoids used in biological control of pest insects.

| **Crop (Country)** | **Pest insect** | **Primary parasitoid** | **Hyperparasitoids^a^** | **Hyperparasitism rate^b^** | **Reference** |
| --- | --- | --- | --- | --- | --- |
| Greenhouse crops | | | | | |
| Sweet pepper^c^ (Canada) | Aphids, e.g.:  *Myzus persicae*  *Aulacorthum solani* | *Aphidius* spp.  *Praon* spp.  *Aphelinus* spp.  And others | *Dendrocerus* spp.  *Asaphes* spp.  *Pachyneuron* spp.  *Alloxysta* spp.  *Syrphophagous* spp. | 8 to 43% (greenhouse)  <10% (field) | 1 |
| Sweet pepper  (The Netherlands) | Aphids, e.g.:  *Myzus persicae*  *Aulacorthum solani*  *Aphis gossypii* | *Aphidius* spp.  *Aphelinus* spp.  *Praon volucre* | *Dendrocerus aphidum*  *Asaphes suspensus*  *Phaenoglyphis villosa* | 18 to 91% | 2 |
| Sweet pepper and egg plant  (Japan) | Aphids (on crop), e.g.:  *Myzus persicae*  *Aphis gossypii* | Barley or wheat bankerplants with *Aphidius* *colemani* on *Rhopalosiphum padi* | *Dendrocerus* spp.  *Asaphes suspensus*  *Pachyneuron* *aphidis*  *Alloxysta* spp.  *Phaenoglyphis villosa*  *Syrphophagous* sp. | <35 to >70% (banker plants)  0 to 100% (crop) | 3 |
| Annual field crops | | | | | |
| Winter wheat, barley and triticale  (France) | Aphids, e.g.:  *Metopolophium dirhodum*  *Rhopalosiphum padi*  *Sitobion avenae* | *Aphidius* spp.  *Ephedrus plagiator*  *Diaeretiella rapae* | *Asaphes* spp.  *Alloxysta* spp.  *Phaenoglyphis* spp. | 0 to 21% | 4  5 |
| Broccoli (U.S.A.) | Cabbage white:  *Pieris rapae* | *Cotesia rubecula*  *Cotesia glomerata* | *Spilochalcis torvina*  *Tetrastichus galactopus*  *Catolaccus aeneouiridis*  *Isdromas lycaenae* | ~8% (*C. glomerata*)  ~38% (*C. rubecula*) | 6 |
| Brussels sprouts  (The Netherlands) | Aphids, e.g.:  *Brevicoryne brassicae*  *Myzus persicae* | *Aphidius* spp.  *Diaeretiella rapae*  *Praon volucre* | *Dendrocerus* spp.  *Asaphes* spp.  *Pachyneuron* *aphidis*  *Coruna clavata*  *Alloxysta* spp.  *Phaenoglyphis villosa*  *Syrphophagous* *aphidivorus* | >80% | 7  8 |
| Cassava (Africa) | Cassava mealybug  *Phenacoccus manihoti* | *Apoanagyrus lopezi* | At least 10 species, e.g.:  *Chartocerus hyalipennis*  *Prochiloneurus* spp.  *Tetrastichus* sp.  *Marietta leopardina* | 20 to 90% | 9-12 |
| Cotton (China) | Aphids:  *Aphis gossypii* | *Binodoxys communis*  *Aphidius gifuensis*  *Aphelinus albipodus* | *Dendrocerus laticeps*  *Asaphes suspensus*  *Pachyneuron* *aphidis*  *Alloxysta* spp.  *Phaenoglyphis villosa*  *Syrphophagous* spp. | 67 to 98% | 13 |
| Leek | Leek moth:  *Acrolepiopsis assectella* | *Diadromus pulchellus* | *Conura albifrons* | NA | 14 |
| Fruit orchards | | | | | |
| Citrus  (Spain) | Aphids:  *Aphis spiraecola* | *Binodoxys angelicae* | *Syrphophagous* *aphidivorus*  *Alloxysta* sp.  *Dendrocerus* sp.  *Asaphes* sp.  *Pachyneuron* *aphidis*  *Phaenoglyphis villosa* | 55 to 81% | 15 |
| Citrus (France) | Armored scales:  *Aonidiella aurantii*  *Unaspis yanonensis* | *Aphytis* spp.  *Encarsia* spp. | *Ablerus* sp. | ~1% | 16 |
| Cultivated forest | | | | | |
| Oak  (Austria) | Gypsy moth Lymantria dispar | *Cotesia melanoscela* (dominant) | 16 species, e.g.:  *Gelis* spp.  *Lysybia nana*  *Mesochorus* sp.  *Pteromalus chrysos*  *Tetrastichus* spp. | ~50% | 17 |

^a^ The main (dominant) hyperparasitoid genera or species are listed per system

^b^ Approximate percentage of primary parasitoids killed by hyperparasitoids

^c^ In this study, hyperparasitism on aphid parasitoids in sweet pepper was monitored in greenhouses as well as in open fields in western Canada.

1. Acheampong S, Gillespie DR and Quiring DJM, Survey of parasitoids and hyperparasitoids (Hymenoptera) of the green peach aphid, *Myzus persicae* and the foxglove aphid, *Aulacorthum solani* (Hemiptera: Aphididae) in British Columbia. *J Entomol Soc B C*  **109**:12-22 (2012).

2. Bloemhard CMJ, van der Wielen M and Messelink GJ, Seasonal abundance of aphid hyperparasitoids in organic greenhouse crops in The Netherlands. *IOBC-WPRS Bulletin*  **102**:15-19 (2014).

3. Nagasaka K, Takahasi N and Okabayashi T, Impact of secondary parasitism on *Aphidius colemani* in the banker plant system on aphid control in commercial greenhouses in Kochi, Japan. *Appl Entomol Zool*  **45**:541-550 (2010).

4. Tougeron K, Damien M, Le Lann C, Brodeur J and van Baaren J, Rapid responses of winter aphid-parasitoid communities to climate warming. *Frontiers in Ecology and Evolution*  **6**: 00173 (2018).

5. Höller C, Borgemeister C, Haardt H and Powell W, The relationship between primary parasitoids and hyperparasitoids of cereal aphids: an analysis of field data. *J Anim Ecol*  **62**:12-21 (1993).

6. McDonald RC and Kok LT, Hyperparasites attacking *Cotesia glomerata* (L.) and *Cotesia rubecula* (Marshall) (Hymenoptera: Braconidae) in Southwestern Virginia. *Biol Control*  **1**:170-175 (1991).

7. Bukovinszky T, van Veen FJF, Jongema Y and Dicke M, Direct and indirect effects of resource quality on food web structure. *Science*  **319**:804-807 (2008).

8. Lefort M, Wratten SD, Cusumano A, Varennes Y and Boyer S, Disentangling higher trophic level interactions in the cabbage aphid food web using high-throughput DNA sequencing. *Metabarcoding and Metagenomics*  **1**:e13709 (2017).

9. Zeddies J, Schaab RP, Neuenschwander P and Herren HR, Economics of biological control of cassava mealybug in Africa. *Agricultural Economics*  **24**: 209-219 (2001).

10. Herren HR and Neuenschwander P, Biological control of cassava pests in Africa. *Annu Rev Entomol*  **36**:257-283 (1991).

11. Sullivan DJ and Völkl W, Hyperparasitism: Multitrophic ecology and behavior. *Annu Rev Entomol*  **44**:291-315 (1999).

12. Iziquel Y and Le Ru B, Influence of hyperparasitism on populations of the encyrtid *Epidinocarsis lopezi*, a parasitoid of *Phenacoccus manihoti* introduced in Congo. *Entomol Exp Appl*  **52**:239-248 (1989).

13. Yang F, Wu YK, Xu L, Wang Q, Yao ZW, Zikic V, Tomanovic Z, Ferrer-Suay M, Selfa J, Pujade-Villar J, Lu YH and Guo YY, Species composition and richness of aphid parasitoid wasps in cotton fields in northern China. *Sci Rep*  **7**(2017).

14. Miall JH, Abram PK, Cappuccino N and Mason PG, Potential impact of the native hyperparasitoid *Conura albifrons* (Hymenoptera: Chalcididae) on the exotic biological control agent *Diadromus pulchellus* (Hymenoptera: Ichneumonidae). *Biocontrol Sci Technol*  **24**:611-624 (2014).

15. Gomez-Marco F, Urbaneja A, Jaques JA, Rugman-Jones PF, Stouthamer R and Tena A, Untangling the aphid-parasitoid food web in citrus: Can hyperparasitoids disrupt biological control? *Biol Control*  **81**:111-121 (2015).

16. Correa MCG, Palero F, Dubreuil N, Etienne L, Hulak M, Tison G, Warot S, Crochard D, Ris N and Kreiter P, Molecular characterization of parasitoids from armored scales infesting citrus orchards in Corsica, France. *BioControl*  **61**:639-647 (2016).

17. Eichhorn O, Experimental studies upon the parasitoid complex of the gypsy moth (*Lymantria dispar* L.) (Lep., Lymantriidae) in lower host populations in eastern Austria. *J Appl Entomol*  **120**:205-212 (1996).
